# Age of Onset and Length of Survival of Queensland Patients with Amyotrophic Lateral Sclerosis: Details of Subjects with Early Onset and Subjects with Long Survival

**DOI:** 10.1159/000528875

**Published:** 2022-12-30

**Authors:** Robert J. Nona, Zhouwei Xu, Gail A. Robinson, Robert D. Henderson, Pamela A. McCombe

**Affiliations:** ^a^Centre for Clinical Research, University of Queensland, Herston, Queensland, Australia; ^b^Department of Neurology, Shanghai Jiao Tong University Affiliated Sixth People's Hospital, Shanghai, China; ^c^Queensland Brain Institute and School of Psychology, University of Queensland, St Lucia, Queensland, Australia; ^d^Department of Neurology, Royal Brisbane and Women's Hospital, Herston, Queensland, Australia

**Keywords:** Amyotrophic lateral sclerosis, Age of onset, Early onset, Long survival

## Abstract

**Introduction:**

The aims of the study were to document the characteristics of amyotrophic lateral sclerosis (ALS) patients in Queensland, to examine factors influencing age of onset, and survival, and to study those with early-onset (<45 years) disease and those with long (>5 years) survival.

**Methods:**

We studied subjects seen at the ALS Clinic at the Royal Brisbane and Women's Hospital. We recorded sex, age of onset, region of onset, length of survival, presence of family history, type of disease, and evidence of cognitive involvement. We analysed the influence of these features on age of onset and survival. We analysed the features of patients with early onset of disease and patients with long survival.

**Results:**

There were 855 ALS patients (505 males) in the cohort. The age of onset was lower in males than females, in patients with a family history of ALS compared to those without, and in patients with spinal onset compared to bulbar onset. Early-onset disease was seen in 10% of patients, and had a greater proportion of males, spinal onset, and classical ALS phenotype compared to late-onset disease. Survival was shorter in females, in patients with bulbar onset, and in patients with classical ALS. Long survival was seen in 18% of patients. Patients with long survival had younger age of onset, greater proportion of males, spinal onset, and fewer patients with classical ALS.

**Conclusion:**

Our study confirms that ALS is more prevalent in males and that spinal onset is more common than bulbar onset. Males have earlier onset but longer survival. We found that overall, patients with classical ALS have worse survival than ALS variants, but some patients who were considered to have classical ALS had long survival. This study confirms the similarity of ALS in our region to ALS in other geographical regions.

## Introduction

Amyotrophic lateral sclerosis (ALS) is a fatal neurodegenerative disorder affecting both upper and lower motor neurons, leading to progressive limb weakness and diaphragmatic dysfunction and death from respiratory failure [1]. Diagnosis of ALS requires evidence of abnormality in upper and lower motor neurons [2, 3, 4]. The first signs of weakness can appear in the bulbar or spinal regions [5]. Family history of ALS is evident in 10–15% of patients [6, 7, 8, 9].

ALS can be divided into subtypes. Most patients have classical ALS, with marked upper and lower motor neuron signs [10]. Some patients with ALS have upper motor neuron dominant (UMN-D) [11, 12] or lower motor neuron dominant (LMN-D) disease [13], based on the predominance of clinical signs [14]. Another ALS variant is flail limb syndrome, which has LMN weakness in the upper limbs (flail arm syndrome) or lower limbs (flail leg syndrome), with functional involvement confined to the flail limb for at least 12 months after onset of symptoms [15]. Progressive muscular atrophy (PMA) and primary lateral sclerosis (PLS) are pure lower and upper motor neuron syndromes, respectively [11, 16, 17, 18, 19].

ALS patients vary in the age of onset of disease, with the peak of incidence usually reported to be in the 6th or 7th decade [20, 21, 22, 23]. However, the range of age of onset is wide. Patients with young onset (<45 years or <40 years) are reported [9, 24, 25, 26]. Patients with older age of onset have also been studied; the cut off for older age of onset has ranged from 65 to 80 years of age [27, 28, 29]. Understanding the features associated with young or old age of onset could be of interest in understanding the risk factors for ALS.

The median survival time for ALS patients is typically 2–5 years [1, 30, 31, 32]; however, survival is variable [33]. Some predictors of survival include age of onset, with early onset (<45 years) being associated with longer survival in ALS [34, 35, 36, 37]. The ALS variants (UMN-D, LMN-D, and flail) are reported to have longer survival compared to classical ALS [11, 13, 15, 19, 38, 39, 40]. Patients with survival >5 years, also referred to as “long survivors”, have been noted in several studies with a prevalence of 14–30% of ALS patients [34, 41, 42]. Patients with short survival have also been examined [43, 44]. Understanding the associated features in these patients could help understand resistance to disease.

In this study, we aimed to describe the features of patients attending our clinic and investigate the factors associated with age of onset and survival. We also aimed to determine the frequency and clinical features of early and late onset and short and long survival.

## Materials and Methods

Subjects are patients with a diagnosis of ALS according to the standard criteria relevant at the time of diagnosis [2, 3, 4]. Patients were seen at the ALS clinic at the Royal Brisbane and Women's Hospital from 2004 to 2022. Patient details were recorded in a database, including date of birth, date of diagnosis, date of death or censoring, region of onset (bulbar or spinal), and disease type (classical ALS, UMN-D, LMN-D, or flail limb). UMN-D patients had weakness with prominent spasticity, brisk reflexes, and pseudobulbar speech [11, 12]. LMN-D patients had marked weakness, muscle wasting, and fasciculations [13, 39], often worse distally, and only minimally increased reflexes; flail limb patients had severe proximal and distal weakness and wasting of upper and lower limbs with reduced reflexes [15]. This cohort does not include patients with PMA (other than flail limb) or PLS which are pure upper and lower motor neuron syndromes [11, 16, 17, 18, 19]. The presence of a family history of ALS in one or more first- or second-degree relatives [45] was recorded. The presence of a family history of neuropsychiatric disease (including dementia and psychosis) was recorded.

We have information about cognition for 196 patients. A total of 180 patients had objective testing of cognitive function. Some had detailed neuropsychological testing that assessed cognition (e.g., current/premorbid intelligence, memory, language, executive functions including fluency, attention), behaviour (apathy), and mood (anxiety, depression). Others completed screening tests with the Addenbrooke's cognitive examination revised (ACE-III R) and the Frontal Assessment Battery, as previously described [46], or with the ACE-III R or Edinburgh cognitive and behavioural ALS screen (ECAS) in the clinic. Some had both forms of testing. In addition, a total of 16 patients were diagnosed on clinical grounds as having cognitive or behavioural involvement. Early age of onset disease was taken to be onset <45 years, as previously described [9, 24, 25, 47], and patients with age of onset <45 years were compared with patients with age of onset 45–75 years and age of onset >75 years.

Survival analysis was performed on subjects who had a record of both the date of disease onset and the date of death or censoring. Patients were grouped into those with short, typical, and long survival. Short survival is defined as patients deceased at <2 years, typical survival is defined as patients deceased at 2–5 years, and long survival is defined as patients deceased or still alive at >5 years. We excluded 2 patients with survival >15 years. The study was approved by the Human Research Ethics Committee of the Royal Brisbane and Women's Hospital.

Data were analysed using GraphPad Prism Software (Biotech, USA, 2021). Characteristics of male patients was compared with female patients, using χ^2^ test. Age of onset across different groups is shown as a median with interquartile range (IQR) and was compared using the Mann-Whitney test. Comparison between early (<45 years), mid (45–75 years), and late (≥75 years) onset ALS was calculated with the χ^2^ test. Survival analysis was performed using log-rank Mantel-Cox test, displayed as Kaplan-Meier curves. Comparison of short (<2 years), typical (2–5 years), and long (>5 years) was calculated with the χ^2^ test. To assess whether sex and region of onset are independent risk factors for survival, multiple regression with Cox proportional hazard analysis was used.

## Results

### Patient Characteristics and Sex Distribution

There were 855 patients in the database. Of these, 680 were deceased. There were 505 male and 350 female subjects (ratio 1.4:1). The region of onset was recorded for 813 patients, of whom 265 (33%) had bulbar onset. 302 patients had lower limb onset, 230 patients had upper limb onset, 12 patients had respiratory or trunk onset, and 4 patients had widespread limb onset. For our analysis, we compared bulbar onset patients with the remainder of patients.

A family history of ALS was documented in 106 (12%) patients. A family history of neuropsychiatric disease was documented in 28 (3%) patients. Disease type was characterised in 812 patients, of whom classical ALS was seen in 700 (86%), UMN-D disease in 46 (6%), LMN-D disease in 39 (5%), and flail limb disease in 28 (3%) subjects: 24 patients had flail arms and 4 patients had flail legs.

Data about cognition are available for 196 patients. Evidence of cognitive/or behavioural impairment was documented in 75 patients. Of these, 59 had objective testing of cognitive function, and 16 had clinically obvious cognitive and/or behavioural changes.

Table 1 shows the sex of the patients with different characteristics. The proportion of males, compared with females, was greater in the spinal onset than bulbar onset group (*p* < 0.0069) and in the flail arm group (*p* < 0.0001). The proportion of females, compared with males, was greater in the flail leg group (*p* < 0.0001). The proportion of males compared with females, family history of ALS (*p* = 0.2545), and other disease variants UMN-D (*p* = 0.0727) and LMN-D (*p* = 0.7575) were not of statistical significance. There was no significant difference in the percentage of males and females between the groups with and without cognitive impairment (*p* = 0.8825).

#### Age of Onset

**Fig. 1:**
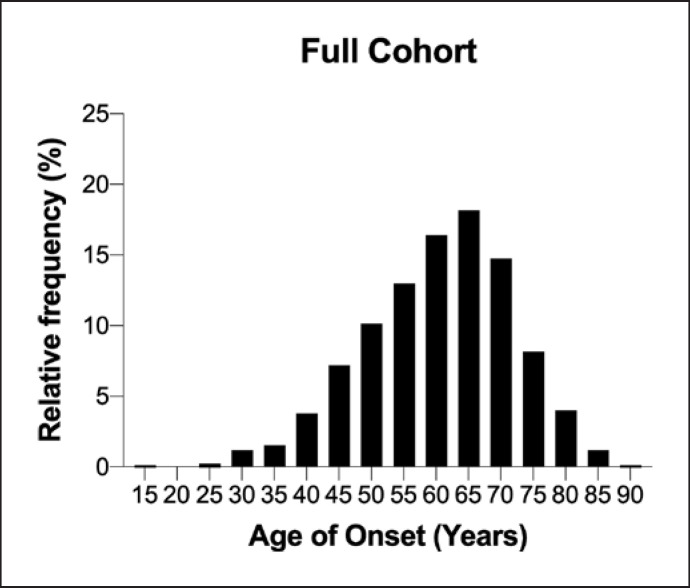
Distribution of the age of onset of disease in patients with ALS. The peak incidence was 65 years. 9% of patients had onset of disease <45 years, and 9.5% of patients had onset >75 years.

**Fig. 2:**
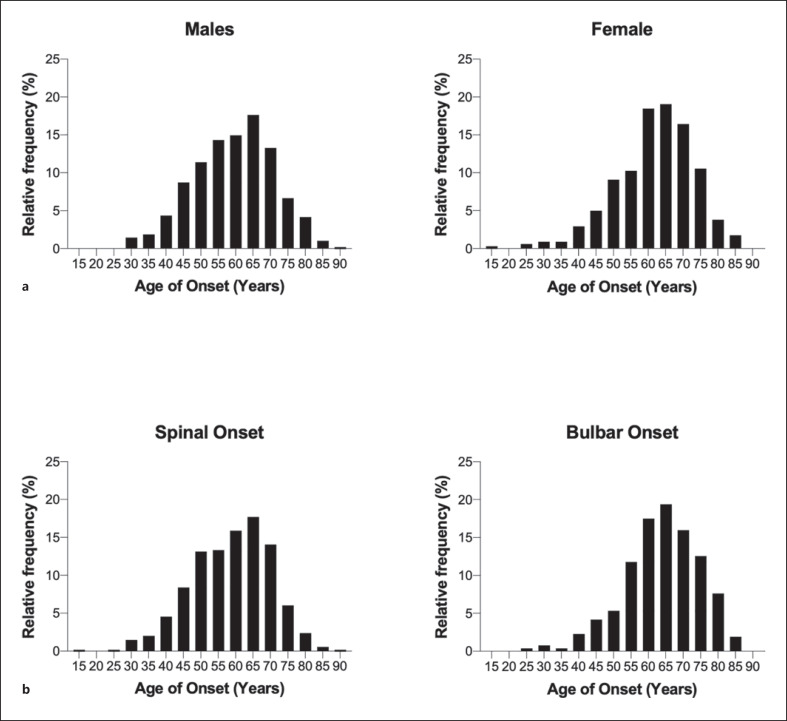
Distribution of age of onset of disease based on the following factors. **a** Sex: median age of onset for males was 60 years and for females was 63 years (*p* = 0.0009, Mann-Whitney test). **b** Region of onset: median age of onset for spinal onset was 60 years, and median age of onset for bulbar onset was 65 years (*p* < 0.0001).

Data about age of onset were available for 823 patients. Table 2 shows the median age of disease onset in different groups. The median (IQR) age of onset for the cohort was 61 years (53–69). The distribution of the age of onset for the entire cohort is shown in Figure 1. The distribution of the age of onset according to sex is shown in Figure 2 (a). The median (IQR) age of onset for males was 60 years (51–68) and for females, 63 years (55–70) (*p* = 0.0009).

**Fig. 3:**
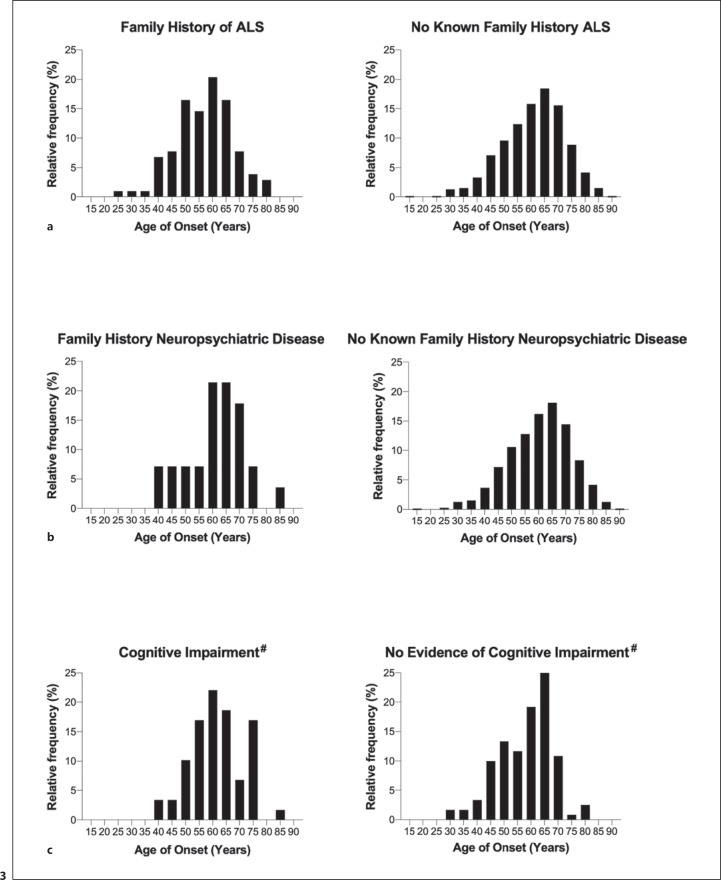
Distribution of age of onset of disease based on the following factors. **a** Family history of ALS: median age of onset was 58 years for patients with a family history of ALS and 62 years with no known family history of ALS (*p* = 0.0004). **b** Family history of neuropsychiatric disease: median age is 63 years for patients with family history of neuropsychiatric disease and 61 with no known family history (*p* = 0.6907). **c** Cognitive impairment: median age of onset was 62 years for patients with formal or clinical evidence of cognitive impairment, and 60 years for patients with no evidence of cognitive impairment.

The distribution of the age of onset, according to region of onset, is shown in Figure 2 (b). The median age of onset (IQR) for bulbar onset was 65 years (57–71) and for spinal onset was 60 years (50–67) (*p* < 0.0001). The distribution of the age of onset according to the presence of a family history of ALS is shown in Figure 3 (a). For patients with a family history of ALS, the median age of onset (IQR) is 58 years (49–64), and for patients without a family history of ALS, it is 62 years (53–69) (*p* = 0.0004). The distribution of the age of onset according to the presence of a family history of neuropsychiatric disease is shown in Figure 3 (b). For patients with a family history of neuropsychiatric disease, the median (age of onset [IQR] was 63 years (55–69), and for patients without a family history of neuropsychiatric disease, it was 61 years (53–69) (*p* = 0.6907).

The distribution of the age of onset according to the presence or absence of cognitive impairment is shown in Figure 3 (c). The median age of onset (IQR) in the group with cognitive involvement was 62 (55–68) years, and for the group with no evidence of cognitive involvement, it was 60 (50–66) years (*p* = 0.0321).

**Fig. 4:**
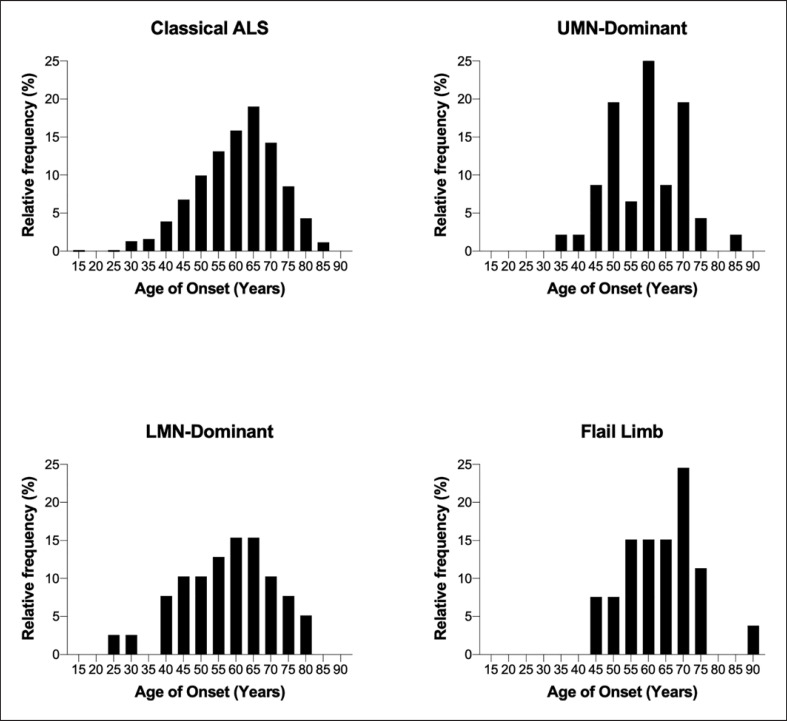
Distribution of age of disease onset based on disease type: median age of onset for classical ALS was 62 years, UMN-D was 60 years (*p* = 0.2348), LMN-D was 59 years (*p* = 0.1874), flail arm was 66 years (*p* = 0.0074), and flail leg was 56 years (*p* = 0.0499).

The distribution of the age of onset, according to type of disease, is shown in Figure 4. The median age of onset (IQR) for classical ALS was 61 years (53–69), UMN-D 60 years (51–68) (*p* = 0.24), LMN-D 59 years (48–67) (*p* = 0.18), flail arm 66 (58–72) (*p* = 0.0074), and flail leg 56 years (49–59) (*p* = 0.05).

#### Early, Mid, and Late Onset Patients

The comparison of patient groups with early, mid, and late onset is shown in Table 3. Early-onset disease (<45 years) was seen in 9% of patients, mid-onset disease was seen in 81.5%, and late-onset disease (>75 years) was seen in 9.5% of patients. The earliest age of onset was 17 years. The latest age of onset was 88 years.

Comparison of the three groups, using χ^2^ analysis, showed significantly more patients with spinal onset (*p* < 0.0001) and more patients with a family history of ALS (*p* = 0.0487) in the early-onset group. The prevalence of cognitive impairment was greatest in the late onset group (*p* = 0.0011).

**Fig. 5:**
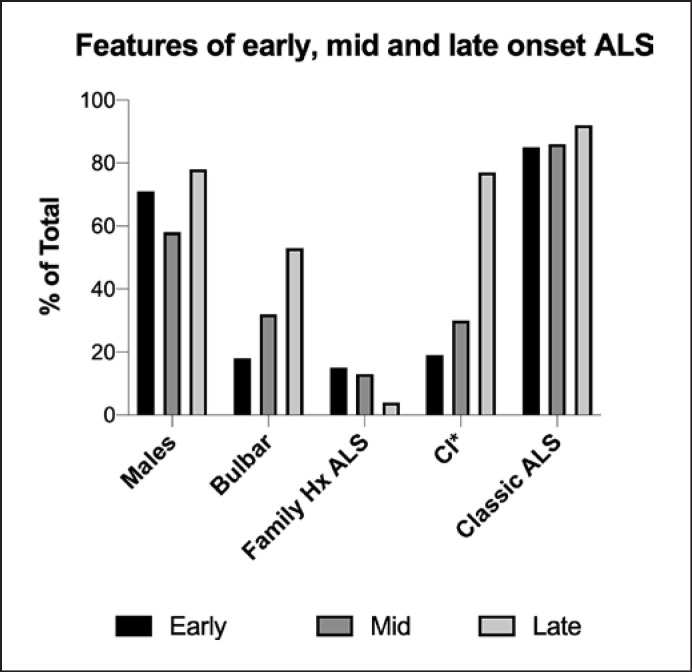
Features of early (<45 years), mid (45–75 years), and late (>75 years) onset ALS. Comparison across the groups using χ^2^ analysis showed the early-onset group had significantly more patients with spinal onset (*p* < 0.0001), more patients with a family history of ALS (*p* = 0.0487), and more patients with of cognitive impairment* (*p* = 0.0011).

In Figure 5, it can be seen that the percentage of males and the percentage of subjects with family history of ALS declines across the three groups. The percentage of subjects with bulbar, the percentage of patients with cognitive impairment, and the percentage of classical ALS increases across the three groups.

#### Survival

Survival data were available for 816 patients, in whom the date of onset and the date of death or censoring were recorded. Of these, 653 patients were deceased. 163 patients were censored after follow-up ranging from 2 months to 18 years from onset of symptoms; the median follow-up was 38 months (20, 68 IQR).

**Fig. 6:**
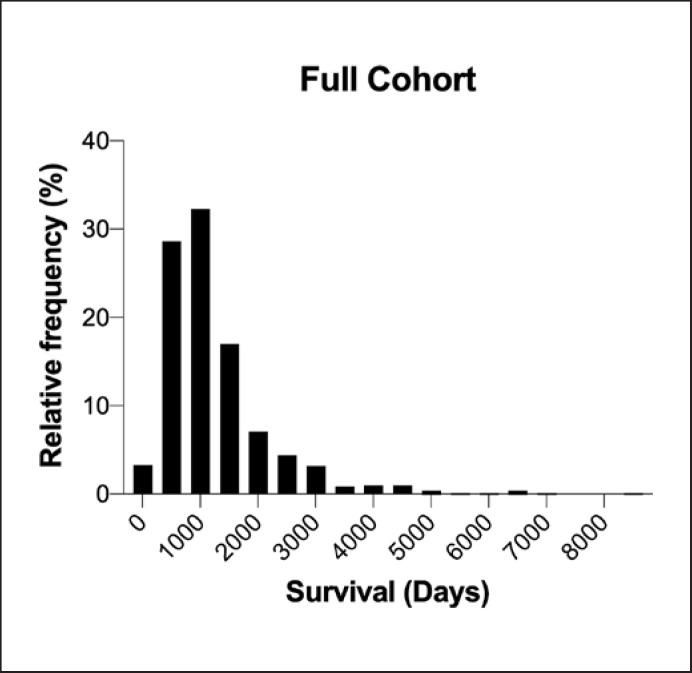
Distribution of survival in the full ALS cohort. It can be seen that the distribution is skewed to the right, with some patients having long survival.

**Fig. 7:**
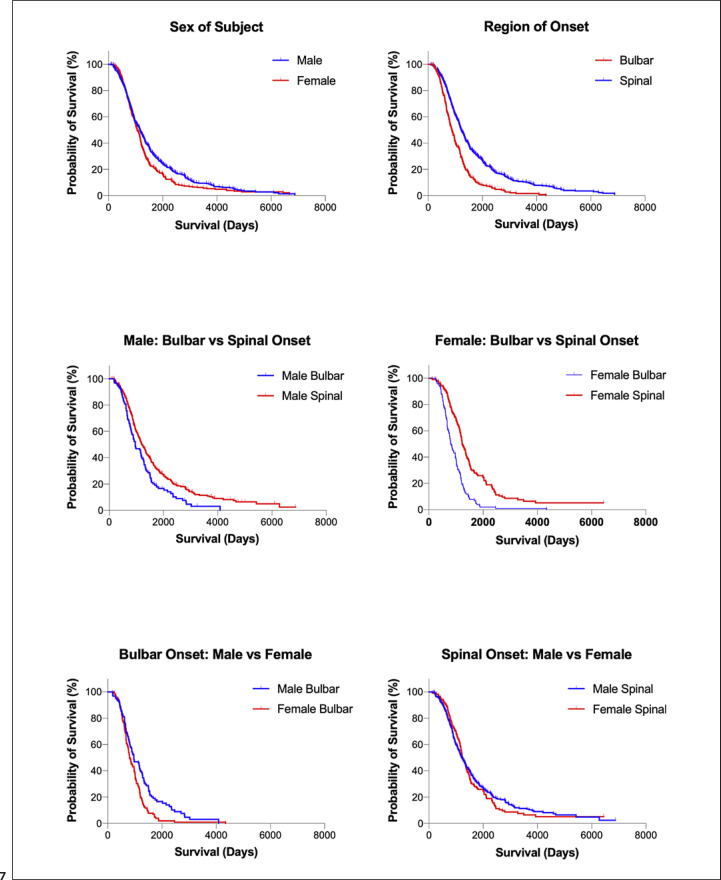
Kaplan-Meier survival curves for ALS cohort: sex and region of onset. Kaplan-Meier survival curves comparing sex and region of onset. Survival in male patients was significantly longer than in female patients. In male patients, survival was significantly longer in those with spinal onset (*p* = 0.0009). In female patients, survival was significantly longer in those with spinal onset (*p* < 0.0001). In bulbar onset patients, survival was longer in males (*p* = 0.0009). In spinal patients, there was no significant difference in survival between males and females (*p* = 0.6683).

**Fig. 8:**
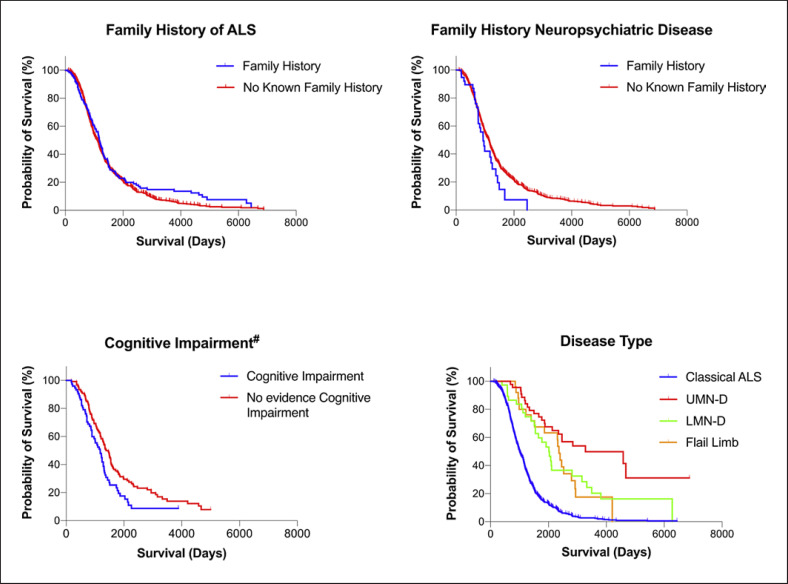
Kaplan-Meier survival curves for ALS cohort: family history, cognitive impairment, and disease type. Survival in patients with classical ALS was shorter than that of patients with UMN-D (*p* < 0.0001), LMN-D (*p* < 0.0001) and flail limb (*p* < 0.0001) and was longer in UMN-D compared with LMN-D (*p* 0.0156), and flail limb (*p* 0.0158). Survival was shorter in patients with cognitive impairment (*p* = 0.0128).

The distribution of length of survival for patients with date of death or censoring is shown in Figure 6. Survival based on clinical features is shown in Tables 4 and 5. Survival curves are shown in Figures 7 and 8. Males have significantly longer median survival than females (*p* = 0.0083). There was significantly longer median survival in patients with spinal onset compared to those with bulbar onset (*p* < 0.0001).

To evaluate the possible interaction of sex and site of onset, we analysed survival according to site of onset in males and females separately. Male subjects with spinal onset had significantly longer survival than male subjects with bulbar onset (*p* = 0.0009). Female subjects with spinal onset had significantly longer survival than female subjects with bulbar onset (*p* < 0.0001). Male subjects with bulbar onset had significantly longer survival than females with bulbar onset (*p* = 0.0009). There is no significant difference in survival between males with spinal and females with spinal onset (*p* = 0.6683). To analyse this further, using multiple regression, we found that when evaluating the effects of sex and region of onset, the hazard ratio for survival of males versus females was 0.867 (*p* = 0.048) and the hazard ration of spinal versus bulbar onset was 0.601 (*p*=<0.001). This shows that much of the difference in survival between males and females is due to the greater prevalence of spinal onset in males.

Compared with classical ALS, there is significantly longer survival for patients with UMN-D (*p* < 0.0001), LMN-D (*p* < 0.0001), and flail limb (*p* < 0.0001) (shown in Table 4; Fig. 5). UMN-D survival was also significantly longer compared with LMN-D (*p* = 0.0156) and flail limb (*p* = 0.0158). Shorter survival was observed in patients with cognitive impairment (*p* = 0.0128). There is no significant difference in survival in patients with family history of ALS compared to those without a family history of ALS (*p* = 0.7091). There was no difference in survival between those with family history of neuropsychiatric disease compared to those without such a history (*p* = 0.0707).

#### Long Survivors

The comparison of short, typical, and long survivor groups is shown in Table 6. Short survival (<2 years) was seen in 31%, typical survival (2–5 years) was seen in 52%, and long survival (>5 years) was seen in 17% of the cohort.

Comparison of the three groups, with χ^2^ analysis, shows the long survivor group had significantly more male patients (*p* = 0.0090), patients with spinal onset (*p* < 0.0001), patients without evidence of cognitive impairment (*p* = 0.0013), and patients with ALS variant disease when compared to classic ALS (*p* ≤ 0.0001). There was no significant difference in the long survivor group in patients with a family history of ALS (*p* = 0.7527), patients with a family history of neuropsychiatric disease (*p* = 0.1637), and comparing patients with ALS variants (*p* = 0.0863–0.8891).

**Fig. 9:**
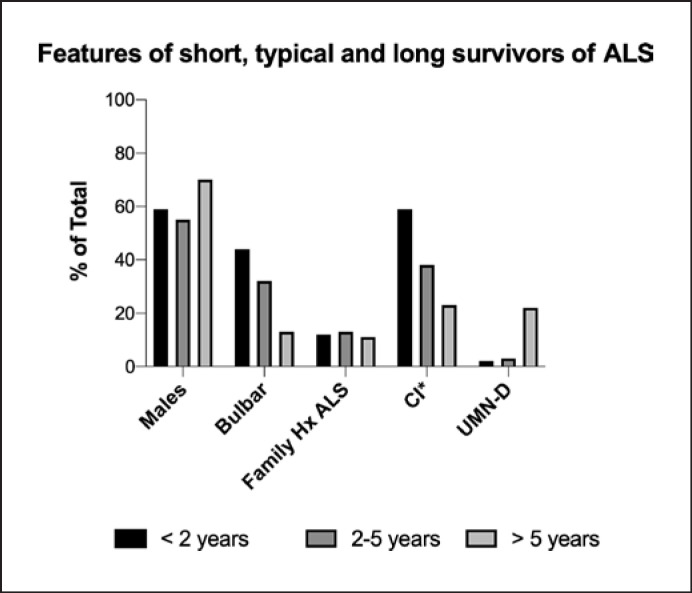
Features of short (<2 years), typical (2–5 years), and long (≥5 years) survivors. Comparison across the groups using χ^2^ analysis, the short survivor group had significantly more male patients (*p* = 0.0090), more patients with spinal onset (*p* < 0.0001), and more patients with cognitive impairment* (*p* = 0.0013). The short survivor group had significantly more patients with UMN-D, LMN-D, and flail, compared with classical ALS (*p* ≤ 0.0001).

Figure 9 shows the percentage of bulbar disease declining across the three groups. The percentage of subjects with UMN-D increases across the three groups. The percentage of cognitive impairment decreases across the three groups.

## Discussion

In this study, we report the clinical features of 855 patients managed at the ALS Clinic in Brisbane, Queensland. We focus particularly on early-onset disease and long survivors. The median age of onset for the cohort was 61 years, consistent with previous epidemiological studies [20, 48, 49, 50, 51]. There was a wide range of survival, with the average survival of the cohort consistent with other studies [20, 22, 23, 24, 30, 35, 52]. We found that 10% of subjects had early onset and 18% had long survival.

We first examined the proportions of males and females in the cohort. There were more males than females, consistent with previous studies [10, 20, 24, 49, 53, 54, 55]. A male predominance is found in other neurodegenerative diseases such as Parkinson's disease [56, 57] and FTD [58, 59, 60]. This male predominance is unexplained but could be related to the effects of genetic factors [61, 62, 63], gonadal hormones [64], sex differences in motor neuron excitability [53, 65], sex differences in the immune system [66, 67], environmental factors [68, 69], and metabolic and stress responses [70].

However, we found that there was a male predominance in patients with spinal onset and a female predominance of bulbar onset, consistent with several studies [5, 24, 48, 71, 72], but the cause of this is not clear. More patients had spinal than bulbar onset, consistent with other series [20, 22, 23, 24, 30, 32, 52]. Although the pathology is similar, the reason why disease starts in a particular region is largely unknown [73, 74, 75]. Pathogenesis is also shown to be similar in bulbar and spinal onset [76, 77]; however, involvement of extra-motor brain regions is greater in bulbar onset [78]. Furthermore, bulbar patients differ from spinal-onset patients in having a greater response to riluzole [79]. However, we did not assess this variable in our cohort.

Next, we examined the age of onset. Males showed a significantly earlier median age of onset than females, similar to other studies [24, 32, 48, 49, 50]. An earlier age of onset of disease in males is also found in Parkinson's disease [56, 80].

Patients with spinal-onset disease had significantly earlier onset than those with bulbar-onset disease. This is comparable with previous studies [9, 24, 25, 47, 49, 81]. Since spinal disease has earlier onset than bulbar disease and since males have more spinal disease than females, it is likely that some of the explanation for earlier onset in males is related to the increased frequency of spinal onset. As expected from the finding of an earlier age of onset in males and in patients with familial ALS, the early-onset group had a greater proportion of males, similar to previous studies [24, 26, 30]. Similarly, the age of onset is lower for patients with spinal disease than bulbar disease, so the proportion of early-onset disease with spinal onset was significantly greater than with bulbar onset, as previously reported.

We also examined length of survival. We found significantly longer survival in male patients, compared to females. This is consistent with other studies showing longer survival in males [34, 35, 82, 83, 84, 85]. Survival in patients with spinal onset was significantly longer than that in those with bulbar onset, consistent with previous studies [34, 35, 82, 83, 84, 85, 86]. Long survival was more prevalent in males and patients with spinal onset, similar to the findings of other [35, 37, 49, 87, 88].

Since more males than females had spinal onset, the increased prevalence of spinal disease could contribute to longer survival in males. With multiple regression analysis, we found that the majority of the difference in survival was due to the site of onset, but there was also a small independent effect of sex. We also found that male subjects with bulbar onset had significantly longer survival than females with bulbar onset (*p* = 0.0009), further indicating that there are sex differences, even after accounting for site of onset.

In this study, we classified patients according to type of disease. This distinction was made so that we could characterise the patients with ALS variants and compare their age of onset and survival with patients with classical ALS. UMN-D disease is recognised in patients who fulfil the criteria for ALS, in having both upper and lower motor neuron signs, but in whom the lower motor neuron signs are not prominent and in whom the denervation, as seen with electromyography, is less marked than in classical ALS [11, 18]. LMN-D disease has prominent lower motor neuron signs, but some evidence of upper motor neuron involvement [13, 39, 89]. Flail limb disease presents with specific features of proximal and distal limb weakness and wasting [15, 90]. The majority of our patients had classical ALS (86%). This is comparable with what is found in the increasing numbers of other studies that have used this classification [11, 18, 23, 39, 91]. The median age of onset of classical ALS patients is consistent with several studies [22, 23, 24, 28, 92]. In comparison with classical ALS, our finding of younger onset in UMN-D, LMN-D, and flail leg disease and later onset in flail arm disease is also consistent with other studies [15, 16, 19, 38, 39, 40, 93].

Patients with UMN-D had significantly longer survival compared with the other disease types, while classical ALS had significantly shorter survival compared with the other disease types. Consistent with this, in the long survivor group, UMN-dominant disease was more frequent than classical ALS, LMN-dominant, and flail limb, as shown in previous studies that reported UMN-D [9, 94]. However, one striking finding is that patients with classical ALS can also have survival ≥5 years from onset.

We have also examined the patients with a family history of ALS. In our series, 12% of patients had a family history of ALS. This is similar to the results of other studies [23, 30, 52], although other studies of ALS cohorts report lower [20, 95] and higher [21, 96, 97] rates of family history of ALS. An earlier age of onset is shown in patients with a family history of ALS, comparable with other reports [9, 97, 98]. It might be expected that patients with a family history of ALS carry increased genetically determined vlunerability to ALS and could therefore have earlier onset [96, 97, 99, 100]. Consistent with this, the early-onset group had significantly more subjects with familial ALS than the late-onset group [6, 8, 101]. We found no significant difference in survival in those with a family history of ALS, which is consistent with other studies [25, 97, 102]. However, there are multiple factors potentially survival in those with family history of ALS [33].

A family history of neuropsychiatric diseases, including schizophrenia [103] and depression [103, 104], has been shown to be higher in ALS subjects compared with controls. A family history of neuropsychiatric disease was not associated with age of onset or survival.

We have looked at the effects of cognitive impairment, although our data are limited. We found that age of onset was later and survival was shorter, in those with evidence of cognitive impairment, which is consistent with other studies [28, 46, 105, 106]. This is reflected in the lower prevalence of cognitive impairment in the subjects with early onset and the lower prevalence of cognitive impairment in the long survivors. We have previously shown that patients with cognitive impairment are known to have more widespread abnormality on MR imaging than those without [107]; this could explain the faster progression and shorter survival.

There are limitations to our study. One limitation is that this is a cohort study rather than a population-based study or a national registry study. Population-based and registry studies [108, 109, 110, 111] have the advantage of complete ascertainment, whereas cohort studies are biased by patterns of referral. This possible bias could influence the estimation of the percentages of patients of different sex, different sites of onset, different types of disease, and the percentage of patients with cognitive impairment. However, the analysis of the age of onset and survival of patients with these features would not be affected. Regarding the analysis of the flail-leg subjects, we acknowledge that the number of these patients was small.

Another issue is that we used clinical judgement to assign patients into UMN-D and LMN-D groups. The relationship of the UMN-D and LMN-D groups to PLS and PMA could be debated, and the general consensus is that there is a spectrum of disease [4, 38, 48]. However, by identifying patients with non-classical ALS, we were able to study the features of patients with classical ALS.

## Conclusion

This study looks closely at the effects of clinical phenotype and sex on the age of onset and survival of patients with ALS. In particular, we classified patients into typical ALS and ALS variants and showed that this affected survival. We also studied early onset and long survival in ALS. The early-onset group had more patients with familial disease, more males, and more spinal-onset disease than the late-onset group. The long survivor group shows that even after removing patients with non-classical ALS, there are significant numbers of patients with classical ALS who have long survival. Exploring these groups is of interest because it would be of great value to know the factors that lead to early onset of disease and long survival. More detailed studies are required to determine the factors that lead to a slower course of disease.

## Statement of Ethics

The study was approved by the Human Research Ethics Committee of the Royal Brisbane and Women's Hospital, including a waiver of consent (approval number HREC/2021/QRBW/79331).

## Conflict of Interest Statement

The authors have no conflicts of interest to declare.

## Funding Sources

No funding was provided in this study.

## Author Contributions

Robert J Nona analysed the data and wrote the first draft of the paper. Zhouwei Xu, Gail Robinson, Robert D Henderson, and Pamela A McCombe provided data and edited the paper.

## Data Availability Statement

All data generated or analysed during this study are included in this article. Further enquiries can be directed to the corresponding author.

## Figures and Tables

**Table 1:** Sex distribution of patients in different groups

	Number males (%)	Number females (%)	Comparison of subject sex (χ^2^)
Total (*n* = 855)	505 (59)	350 (41)	−
Region of onset (*n* = 813)			
Bulbar onset	123 (46)	142 (54)	**0.0069**
Spinal onset	356 (65)	192 (35)	
Family history (*n* = 855) ALS			
Family history	55 (52)	51 (48)	0.2545
No known family history	450 (60)	299 (40)	
Neuropsychiatric disease			
Family history	17 (61)	11 (39)	0.0792
No known family history	488 (59)	339 (61)	
Cognitive impairment (CI) ^a^ (*n* = 196)			
Evidence of CI	49 (65)	26 (35)	0.9013
No evidence of CI	78 (64)	43 (36)	
Disease type (*n* = 812)			
Classical ALS	406 (57)	294 (43)	0.0727*
UMN-dominant	27 (59)	19 (41)	
LMN-dominant	23 (59)	16 (41)	0.7575*
Flail arm	19 (83)	5 (17)	**<0.0001** *
Flail leg	1 (25)	3 (75)	**<0.0001** *

* Comparison with classical ALS.

a Formal cognitive assessment or clinical cognitive impairment of frontotemporal type.

**Table 2:** Median age of onset in ALS cohort

	Median (IQR) age of onset, years	*p* value (Mann-Whitney)
Total cohort (*n* = 823)	61 (53, 69)	−
Sex (*n* = 823)		
Males	60 (51, 68)	**0.0009**
Females	63 (55, 70)	
Region of onset (*n* = 811)		
Bulbar onset	65 (57, 71)	**<0.0001**
Spinal onset	60 (50, 67)	
Family history (*n* = 823) ALS		
Family history	58 (49, 64)	**0.0004**
No known family history	62 (53, 69)	
Neuropsychiatric disease		
Family history	63 (55, 69)	0.6907
No known family history	61 (53, 69)	
Cognitive impairment (CI) (*n* = 195)^a^		
Evidence of CI	62 (55, 68)	**0.0321**
No evidence of CI	60 (50, 66)	
Disease type (*n* = 806)		
Classical ALS	62 (53, 69)	0.2348*
UMN-dominant	60 (51, 68)	
LMN-dominant	59 (48, 67)	0.1874*
Flail arm	66 (58, 72)	**0.0074** *
Flail leg	56 (49, 59)	**0.0499** *

IQR, interquartile range.

* Comparison with classical ALS.

a Cognitive assessment or clinical cognitive impairment of frontotemporal type.

**Table 3:** Features of early, mid, and late onset ALS

	Early onset <45 years, *N* (%)	Mid onset 45–74 years, *N* (%)	Late onset ≥75 years, *N* (%)	*p* value (χ^2^)
Sex	*n* = 75	*n* = 669	*n* = 78	
Male	53 (71)	385 (58)	44 (53)	0.0837
Female	22	284	34	
Region of onset	*n* = 74	*n* = 662	*n* = 74	
Bulbar onset	13 (18)	211 (32)	39 (53)	**<0.0001**
Spinal onset	61	451	35	
Family history ALS	*n* = 75	*n* = 669	*n* = 78	
Family history	11 (15)	89 (13)	3 (4)	0.0487
No known family history Neuropsychiatric condition	64	580	75	
Family history	2 (3)	23 (3)	3 (3)	0.9173
No known family history	73	646	75	
Cognitive impairment (CI)^a^	*n* = 16	*n* = 148	*n* = 13	
Evidence of CI	3 (19)	44 (30)	10 (77)	**0.0011**
No evidence of CI	13	104	3	
Disease type	*n* = 74	*n* = 655	*n* = 75	
Classical ALS	63 (85)	562 (86)	69 (92)	0.3092
Non-classical ALS*	11	93	6	

* Comprising UMN-dominant, LMN-dominant, and flail limb subjects.

a Formal cognitive assessment or clinical cognitive impairment ol frontotemporal type.

**Table 4:** Survival in different patient groups

	Median (95% CI) survival days	*p* value (Mantel-Cox)
Sex (*n* = 816)		
Male (478)	1,165 (0.9598–1.268)	**0.0083**
Female (338)	1,056 (0.7886–1.042)	
Region of onset (*n* = 805)		
Bulbar onset (262)	873 (0.615–0.8259)	**<0.0001**
Spinal onset (543)	1,225 (1.211–1.626)	
Family history (*n* = 816) ALS		
Family history (102)	1,181 (0.8561–1.296)	0.7091
No known family history (714)	1,121 (0.7714–1.168)	
Neuropsychiatric condition		
Family history (28)	936 (0.5627–1.196)	0.0707
No known family history (788)	1,141 (0.8362–1.777)	
Cognitive impairment (CI) (*n* = 194)^a^		
Evidence of CI (74)	1,175 (0.6148–1.174)	**0.0128**
No evidence of CI (120)	1,383 (0.8517–1.627)	
Disease type (*n* = 802)		
Classical ALS (691)	1,011 (0.2015–0.4723)	
UMN-dominant (46)	3,277 (2.117–4.962)	<0.0001*
		**0.0156** ^b^
		**0.0158** ^c^
LMN-dominant (38)	2017 (1.356–2.935)	<0.0001*
		0.9836^c^
Flail limb (27)	2368 (1.483–3.699)	<0.0001*

a Formal cognitive assessment and clinical cognitive impairment of frontotemporal type.

* Comparison with Classical ALS

b LMN-D

c flail limb.

**Table 5:** Survival comparing sex and region of onset

	Males median survival (IQR)	Females median survival (IQR)	*p* value (Mantel-Cox)
	*n* = 122	*n* = 160	
Bulbar onset	961 (0.9598**−**1.268)	807 (0.5162**−**0.8311)	**0.0009**
	*n* = 352	*n* = 193	
Spinal onset	1,225 (1.014**−**1.602)	1,232 (1.203**−**1.937)	0.6683
*p* value (Mantel-Cox)	**0.0009**	**<0.0001**	

**Table 6:** Features of patients with short, typical and long survival

	<2 years *n* (%)	2–5 years *n* (%)	≥5 years^a^ *n* (%)	*p* value (χ^2^)
Sex	*n* = 251	*n* = 426	*n* = 135	
Male	147 (59)	234 (55)	94 (70)	**0.0090**
Female	103	192	41	
Region of onset	*n* = 246	*n* = 420	*n* = 134	
Bulbar onset	108 (44)	136 (32)	18 (13)	**<0.0001**
Spinal onset	139	284	116	
Family history ALS	*n* = 251	*n* = 426	*n* = 135	
Family history	31 (12)	54 (13)	15 (11)	0.7527
No known family history	220	372	120	
Neuropsychiatric condition				
Family history	10 (4)	17 (4)	1 (<1)	0.1637
No known family history	241	372	120	
Cognitive impairment (CI)^b^	*n* = 44	*n* = 96	*n* = 56	
Evidence of CI	26 (59)	36 (38)	13 (23)	**0.0013**
No evidence of CI	18	60	43	
Disease type	*n* = 246	*n* = 418	*n* = 134	
Classical ALS	237 (96)	381 (91)	71 (53)	
UMN-dominant	2	13	30	**<0.0001** *
				0.0863** 0.6902^c^ 0.8891^d^
LMN-dominant	6	14	17	**<0.0001** *
				0.3613^c^ 0.4877^d^
Flail arm	1	9	13	**<0.0001** *
				0.7581^d^
Flail leg	0	1	3	**0.0001** *

a Upper limit of 15 years for cohort analysis.

b Cognitive assessment or clinical cognitive impairment of frontotemporal type.

* Comparison with classical ALS

** LMN-D

c flail arm

d flail leg.
